# Modulating subjective and objective cognitive state fatigue in long COVID with repetitive anodal tDCS: results from a double-blinded randomized controlled trial

**DOI:** 10.1186/s12868-025-00989-x

**Published:** 2026-01-24

**Authors:** Magdalena Mischke, Tino Zaehle

**Affiliations:** 1 Departments of Neurology, Otto-von-Guericke University, Leipziger Straße 44, 39120 Magdeburg, Germany; 2 Institute for Medical Psychology, Otto-von-Guericke University, Leipziger Straße 44, 39120 Magdeburg, Germany; 3 German Center for Mental Health (DZPG), Partner Site Halle-Jena-Magdeburg, Magdeburg, Germany; 4 Center for Behavioral Brain Sciences, Magdeburg, Germany; 5 Institute for Medical Psychology & Clinic for Neurology, Otto-von-Guericke University, Leipziger Straße 44, 39120 Magdeburg, Germany

**Keywords:** Long covid, Cognitive fatigue, Subjective and objective state fatigue, Transcranial direct current stimulation, EEG

## Abstract

**Background:**

Cognitive fatigue is a frequently reported and debilitating symptom of long COVID, yet effective therapeutic interventions remain limited. Anodal transcranial direct current stimulation (tDCS) over the dorsolateral prefrontal cortex (dlPFC) has been proposed as a promising approach to modulate fatigue-related neural networks. To comprehensively assess cognitive fatigue, the integration of subjective and objective behavioral and electrophysiological measures of induced state fatigue is essential.

**Methods:**

This double-blind, randomized, sham-controlled study investigated the effects of four consecutive daily sessions of 30-minute anodal tDCS over the left dlPFC on subjective and objective markers of cognitive state fatigue in individuals with long COVID. The present paper focuses on secondary outcomes, including subjective state fatigue ratings via visual analogue scales, behavioral performance indices, and electrophysiological markers such as temporal alterations of frontal theta and occipital alpha activity as well as p50 sensory gating.

**Results:**

Forty participants received either verum or sham tDCS while completing a gamified adaptive Go/No-Go task. Before and after the stimulation period, cognitive state fatigue was reliably induced using the AX-Continuous Performance Task (AX-CPT). Although tDCS did not significantly affect subjective state-fatigue ratings or behavioral performance, our findings indicate that verum stimulation may stabilize fatigue-related changes in occipital alpha power. No immediate stimulation-related improvements were found in the Go/No-Go task.

**Conclusions:**

These findings indicate that while tDCS may modulate neurophysiological correlates of cognitive state fatigue, its impact on subjective experience and behavioral performance remain limited under the current protocol. These results, however, underscore the importance of including neurophysiological endpoints in intervention research and highlight the need for developing more robust and individualized stimulation protocols. Future studies should consider extended stimulation regimens, alternative task paradigms, and more sensitive behavioral measures to further elucidate the neuromodulatory potential of tDCS in long COVID-related cognitive fatigue.

**Trial registration:**

drks.de Identifier: DRKS00031294, date of registration: 17.02.2023

## Background

The initial emergence of SARS-CoV-2 in Wuhan, China, and its rapid global spread led the World Health Organization (WHO) to declare COVID-19 a pandemic in March 2020 [1, 2]. This unprecedented global health crisis not only strained healthcare systems but also introduced significant disruptions to daily life through widespread lockdowns [3]. As the acute phase of the pandemic progressed, a growing number of individuals began to report persistent symptoms following their initial infections, now recognized as long COVID [4]. The WHO defines long COVID as the continuation or emergence of new symptoms three months post-infection, lasting for at least two months and not attributable to alternative diagnoses [5]. Terms such as “post-acute sequelae of SARS-CoV-2 infection” and “post COVID” have been used interchangeably; however, this paper will consistently refer to it as long COVID. Long COVID presents a multifaceted clinical picture, affecting various organ systems and manifesting in persistent respiratory, cardiovascular, and neuropsychological symptoms [6]. Among these, fatigue stands out as one of the most prevalent and distressing complaints [7, 8]. Characterized by debilitating exhaustion that is exacerbated by physical activity and unrelieved by rest or sleep, fatigue in long COVID resembles symptoms observed in neurodegenerative disorders such as Multiple Sclerosis (MS), Parkinson’s disease, and Myalgic Encephalomyelitis/Chronic Fatigue Syndrome (ME/CFS) [9–12]. Although some patients may experience partial remission over time, fatigue often becomes chronic, severely impairing daily activities and diminishing quality of life, leading to early retirement and mental health challenges [13, 14].

A major limitation in the diagnosis and study of fatigue is its inherently subjective character; current assessments rely predominantly on self-reported measures or questionnaire-based evaluations, which tend to conceptualise fatigue as a stable trait [15]. Given that long COVID remains a diagnosis of exclusion, and fatigue—a cardinal symptom—relies predominantly on subjective self-reports, affected individuals frequently encounter underrecognition of their symptoms, a lack of objective diagnostic markers, and extended intervals without adequate medical support [16, 17].

To account for the multifaceted nature of fatigue, we proposed a comprehensive model that distinguishes between cognitive trait fatigue and both subjective and objective state components [18]. Subjective state fatigue refers to the momentary perception of mental exhaustion, typically modulated by task demands, and is commonly quantified using visual analogue (VAS) or numerical rating scales (NRS). In contrast, objective state fatigue - often also referred to as fatigability - captures quantifiable declines in performance and electrophysiological alterations over time [18].

Individuals with long COVID frequently show deficits in executive functioning [19], manifested by reduced accuracy, impaired inhibitory control and prolonged reaction times during attentional tasks, relative to healthy controls alongside elevated levels of subjective fatigue [20, 21]. Electrophysiological correlates pertinent to transdiagnostic fatigue research - and indicative of fatigability - include frontal theta activity, alpha activity over task-relevant cortical regions, and auditory p50 sensory gating [22, 23]. Frontal theta activity is considered a compensatory mechanism supporting performance maintenance during sustained attention tasks. Frontal theta increases with rising demands of executive functioning [22–24]. In contrast, alpha power tends to rise progressively during task engagement in association with the development of state fatigue [24–26], a pattern that appears more pronounced in individuals experiencing pathological fatigue [23]. Auditory p50 sensory gating, reflecting thalamocortical top-down control mechanisms that mitigate sensory overload [27], has been shown to be impaired under experimentally induced state fatigue, notably more prominent in individuals with MS [22].

The pathophysiological mechanisms underlying long COVID related fatigue remain incompletely understood [6]. Current hypotheses on cognitive fatigue implicate a combination of neuroimmunological factors, structural and functional brain alterations, dysregulation of cerebral perfusion, and compromised blood-brain barrier integrity [6, 10, 28]. Acute SARS-CoV-2 infection is known to provoke an exaggerated immune response, leading to sustained production of pro-inflammatory cytokines and chronic inflammation [29–31]. Evidence of neuroinflammation in long COVID includes the infiltration of activated immune cells and cytokines into the central nervous system, which may trigger microglial activation and compromise vascular integrity [32–34]. Increased permeability of the blood-brain barrier and structural abnormalities in brain regions associated with cognitive functions - particularly in frontal areas - have been observed in this population [28, 35, 36]. Notably, these alterations may result from direct viral neurotropism, hypoxic–ischemic injury, or secondary systemic inflammatory processes, and are thought to contribute to frontal hypoactivity frequently reported in long COVID [10]. Frontal hypoactivity, in turn, is hypothesized to disrupt the cortico-striato-thalamo-cortical network - a neural circuit closely linked to fatigue [10, 37]. Consistent with this, patients with long COVID often exhibit executive function impairments that mirror patterns of reduced prefrontal activation [20]. At present, therapeutic strategies for long COVID-related fatigue remain largely symptomatic. Most proposed interventions are derived from case series or early-stage pilot studies and lack robust empirical validation in controlled clinical trials [6].

In light of the current lack of effective treatments long COVID related fatigue, transcranial direct current stimulation (tDCS) emerges as a promising therapeutic strategy for targeting frontal hypoactivity [10]. tDCS is a non-invasive neuromodulation technique that delivers a low-intensity direct current via scalp-mounted electrodes to modulate cortical excitability by altering neuronal membrane potentials [38, 39]. Anodal tDCS has been shown to enhance cerebral blood flow in humans [40, 41], and to increase blood-brain barrier permeability in animal models [42], potentially facilitating neurovascular and neuroimmune modulation.

Anodal stimulation over frontal regions, particularly the dorsolateral prefrontal cortex (dlPFC), has demonstrated beneficial effects on subjective fatigue, cognitive performance, and both subjective and objective markers of state fatigue in both healthy individuals [43–47], and patients with MS-related fatigue [45, 48–50]. Preliminary studies investigating tDCS in long COVID-associated fatigue have yielded mixed outcomes probably due to methodological variations in stimulation protocols [51–54]. Compelling evidence from prior work suggests that repetitive applications of tDCS may yield cumulative neuromodulatory effects [55, 56], while online stimulation - administered concurrently with cognitive tasks - has been associated with improved outcomes [45, 57].

The present study reports secondary outcomes from a double-blind, randomized and sham-controlled trial, that investigates the efficacy of repetitive anodal tDCS in alleviating trait and state fatigue associated with long COVID by targeting presumed dysfunctions in the fronto-striato-thalamo-cortical network. Anodal tDCS was applied to the left dlPFC over four consecutive days. The primary outcome - changes in subjective trait fatigue - has been reported elsewhere [58]. While subjective questionnaire outcomes and trait-level electrophysiological data were presented previously [58], in the present paper, we specifically focus on the effects of repeated anodal tDCS on both subjective and objective state fatigue, analyzing specifically fatigue trajectories over time-on-task across verum and sham stimulation conditions. This study including its primary and secondary outcomes was pre-registered with the German Clinical Trials Register (DRKS) under the registration number DRKS00031294. In addition, in the present study, we report data from the gamified adaptive Go/No-Go task administered during the intervention, the analysis of which was not part of the pre-registration.

## Materials and methods

### Design and settings

This pre-registered double-blinded randomized controlled trial was conducted at the Department of Neurology, Otto-von-Guericke University, Magdeburg, Germany, from January to September 2023. This study followed the CONSORT guidelines and was approved by Ethics Committee of the University of Magdeburg. No deviations from the prespecified study protocol registered with the DRKS occurred, except for an extension of the initial recruitment phase by two months, which was updated accordingly in the registry.

### Sample

**Fig. 1:**
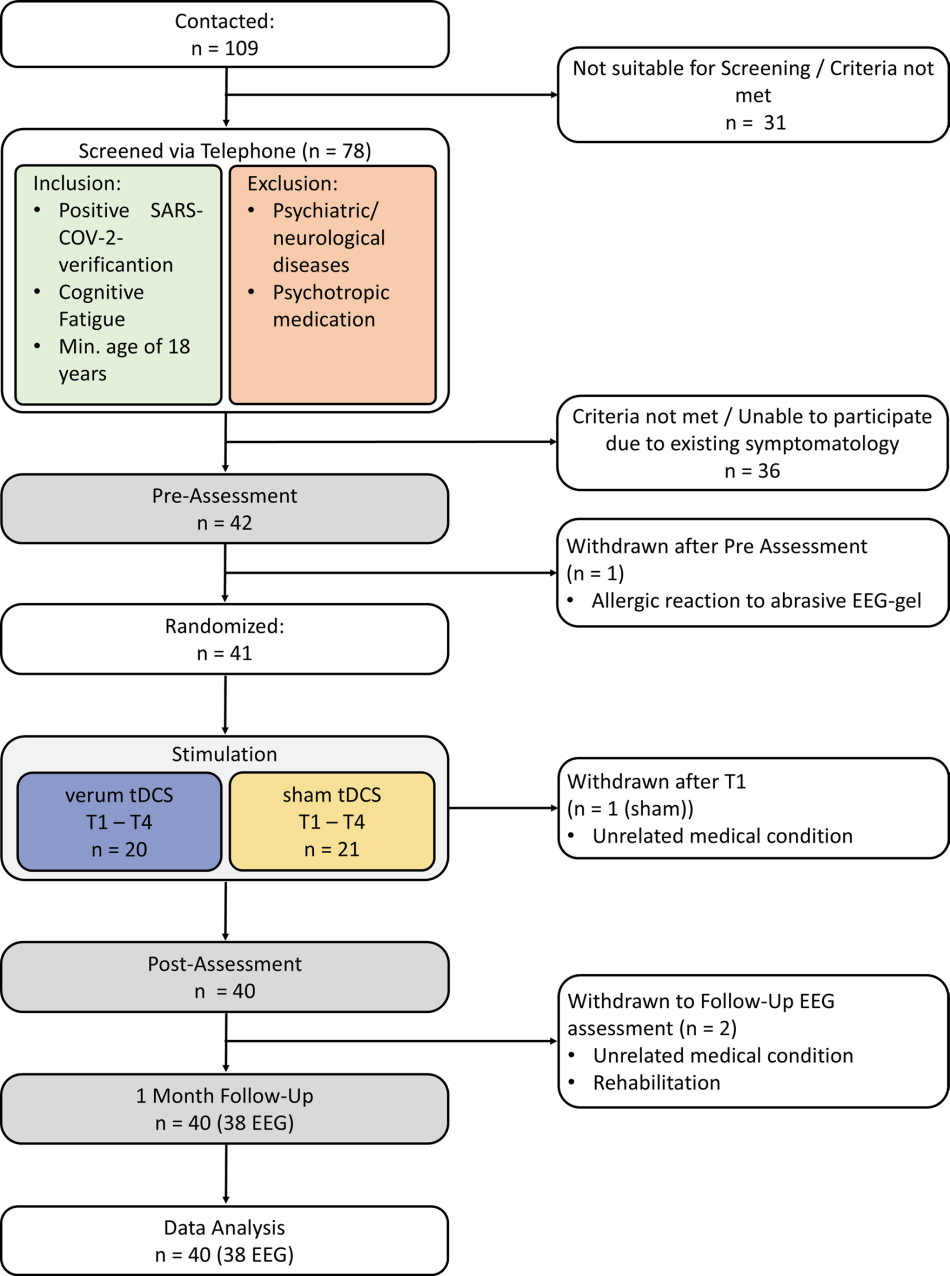
Consort flow diagram showing inclusion and participation of participants throughout the study

A total of 42 individuals with long COVID were recruited. Of these, 41 participants were randomly assigned to receive either active or sham transcranial direct current stimulation (tDCS). Data from 40 participants were included the final analysis (see Fig. 1). Allocation to stimulation condition was predetermined by an independent researcher uninvolved in any subsequent study procedures using block randomization with a block size of ten and an allocation ratio of 1:1. Each participant received a unique identification code linked to their assigned condition, ensuring full blinding throughout the trial.

Prior power analysis for primary outcomes using G*Power indicated that the available sample was sufficient to detect medium-sized effects (Cohen’s *f* = 0.25, *α* = 0.05, statistical power (1–*β*) = 0.90) [59]. Inclusion criteria required a confirmed SARS-CoV-2 infection, a minimum age of 18 years, and a score above 17 on the cognitive subscale of the Würzburger Erschöpfungs-Inventar bei Multipler Sklerose (WEIMuS), indicating clinically relevant cognitive fatigue [60]. Exclusion criteria included acute neurological or psychiatric conditions and current use of psychotropic medications. All participants met standard safety requirements for tDCS application, including absence of seizure history, metallic implants, and cardiac arrhythmias [61]. Eligibility was conducted via telephone prior to enrolment.

In the verum group, participants had a mean age of 49.59 years (SD = 11.75), comprising 7 men and 13 women. The sham group had a comparable age of 49.54 years (SD = 12.36), with 5 men and 15 women. Comprehensive demographic information is presented in Table 1 [58]. Both groups exhibited clinically relevant levels of cognitive fatigue at baseline, with mean scores on the cognitive subscale of the Modified Fatigue Impact Scale (MFIS) of 26.70 (SD = 2.92) in the verum group and 26.05 (SD = 4.72) in the sham group [62]. There were no significant baseline differences between the groups, with two exceptions. First, the time since initial SARS-CoV-2 infection was significantly longer in the verum group (*M* = 19.90 months, SD = 7.99) than in the sham group (*M* = 13.35 months, SD = 6.80), *t*(38) = −2.79, *p* = 0.008. Second, WEIMuS cognitive subscale at screening - used as part of the inclusion criteria - were significantly higher in the verum (*M* = 25.60, SD = 3.65) than in the sham group (*M* = 22.45, SD = 3.12), *t*(38) = −2.93, *p* = 0.006). All ambulatory appointments took place at the university hospital Magdeburg. The study was approved by the Ethics Committee of the University of Magdeburg. All participants gave written informed consent in accordance with the Declaration of Helsinki and received a compensation of €100 for their participation.

**Table 1:** Baseline group characteristics at pre-assessment, mean (sd)

	Total (*n* = 40)	Verum (*n* = 20)	Sham (*n* = 20)	t-value (df)	p-value
Gender [f/m]	28/12	13/7	15/5	χ^2^ (1) = 0.12	0.730
Age [years]	49.56 (11.91)	49.59 (11.75)	49.54 (12.36)	0.01 (38)	0.990
Time to initial infection [months]	16.63 (8.03)	19.90 (7.99)	13.35 (6.80)	2.79 (38)	0.008*
WEIMuS cognitive Subscore [points]	24.03 (3.40)	25.60 (3.65)	22.45 (3.12)	2.93 (38)	0.006*
MFIS Cognitive Subscore [points]	26.38 (3.89)	26.70 (2.92)	26.05 (4.71)	0.52 (38)	0.603
BDI-II [points]	16,88 (6.15)	16,70 (4.43)	17,05 (7.61)	0.18 (38)	0.860
EQ-5D-5L Scale [points]	54,10 (19.87)	51.05 (19.01)	57.15 (20.72)	0.97 (38)	0.338
EQ-5D-5L Index [points]	0.66 (0.27)	0.64 (0.26)	0.69 (0.28)	0.59 (38)	0.562

*WEIMuS*, Würzburger Erschöpfungs-Inventar bei Multipler Sklerose; *MFIS*, Modified Fatigue Impact Scale; *BDI-II*, Becks Depression Inventory II; *EQ-5D-5L*, European quality of life 5 dimensions 5 level version

### tDCS design

This trial comprised four consecutive sessions of ambulatory, daily tDCS, administered by trained study personnel. Stimulation was delivered using the DC-Stimulator Plus (neuroCare Group AG, Munich, Germany). Participants assigned to the active stimulation group received 30 minutes of anodal tDCS per session. The current intensity was ramped up to 1.5 mA over 15 seconds at session onset, maintained for the full stimulation period, and ramped down over an additional 15 seconds. In the sham condition the same ramp-up and ramp-down phases were applied, mimicking the initial cutaneous sensations of active tDCS. Electrode placement was guided by electric field modeling using SimNIBS version 2.1 [63]. Following previous works investigating tDCS effects on cognitive fatigue [38, 50, 64], conductive sponge electrodes soaked in saline solution were used, with the anode (5 × 5 cm^2^) positioned at F3 and the cathode (5 × 7 cm^2^) at F4, according to the international 10–20 EEG system. The bicephalic positioning was chosen to enhance cortical excitability over frontal areas involved in attentional control and executive functioning [24, 38], in order to improve cognitive effort allocation during sustained attention [65–67]. This configuration is intended to counteract frontal hypoactivity and thereby positively influence fronto-striato-thalamo-cortical networks implicated in fatigue regulation [18, 24]. Electrode impedance was monitored throughout all sessions and maintained below 10 kΩ. To potentially augment neuromodulatory effects [45, 57], all participants performed an adaptive, gamified Go/No-Go task during stimulation. Following each session, participants completed a standardized side effect questionnaire to document any adverse events or discomfort [68]. Based on previously published data regarding safety, detailed information on the incidence and intensity of side effects, previously reported elsewhere, is provided in Table 2 [58].

**Table 2:** Mean intensity of side effects experienced during all four tDCS-sessions (0/not at all − 3/severe) with incidence of reportings > 0 in brackets

	Verum (*n* = 20)	Sham (*n* = 20)	*p*-value
Tingling	0.86 (80%)	0.34 (45%)	0.010
Itching	0.69 (65%)	0.24 (40%)	0.031
Headache	0.83 (70%)	0.63 (65%)	0.498
Sleepiness	1.43 (95%)	1.11 (85%)	0.214
Dizziness	0.45 (50%)	0.33 (35%)	0.306
Nausea	0.15 (30%)	0.10 (15%)	0.326
Skin Redness	0.51 (60%)	0.05 (10%)	< 0.001
Skin Irritation	0.13 (30%)	0.01 (5%)	0.039
Heat under the Electrode	0.44 (50%)	0.19 (35%)	0.265
Higher Concentration	0.75 (80%)	0.68 (50%)	0.311

Mean intensity = Sum(side effect intensity) / n, *p*-Values of Wilcoxon rank sum tests comparing intensities of experienced side effects

### Design and clinical evaluation

**Fig. 2:**
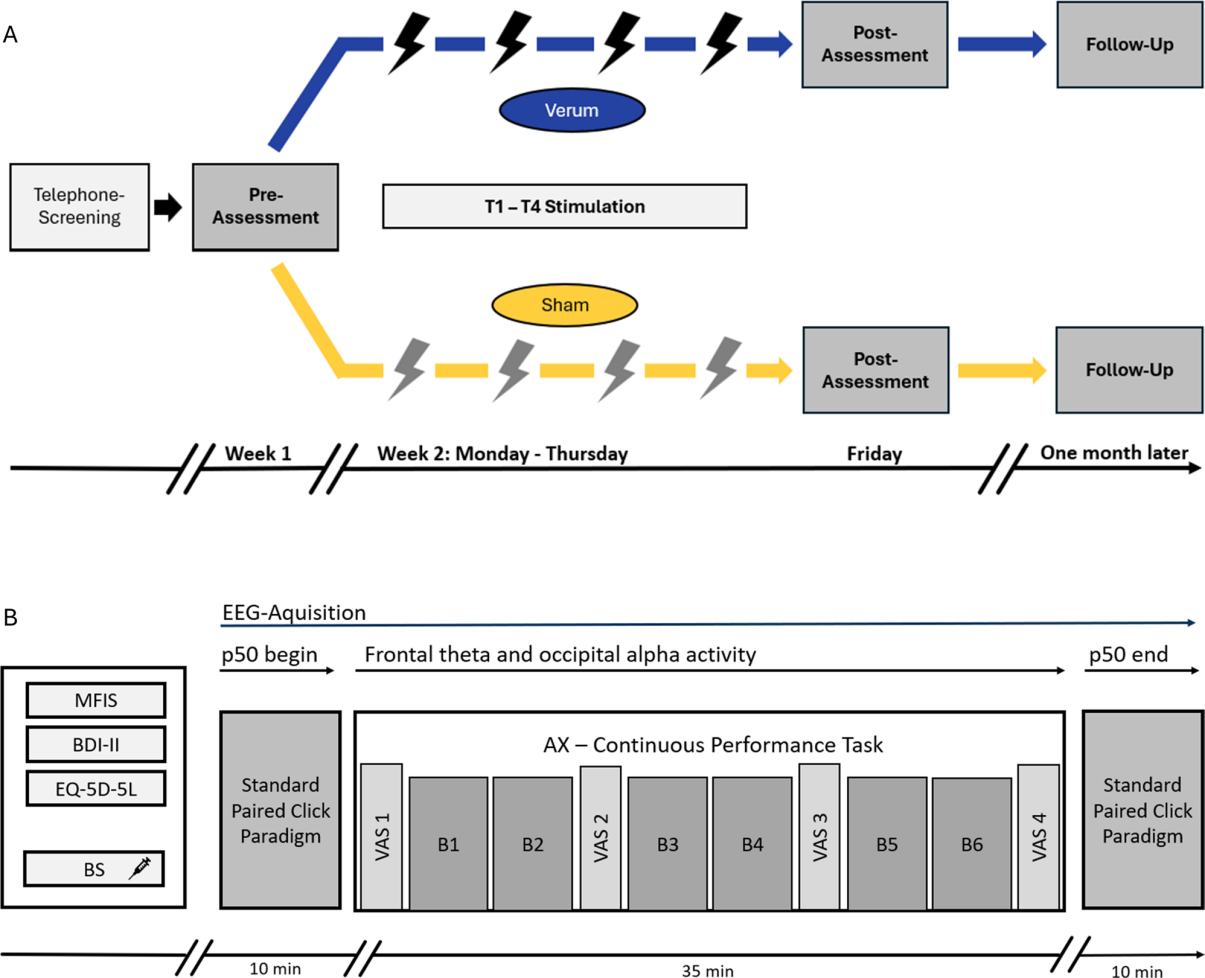
**A**) study design and **B**) design of ambulatory EEG-Assessment (Modified fatigue Impact Scale, MFIS; Becks Depression Inventory ii, BDI-II; European quality of life 5 dimensions 5 level version, EQ-5D-5L; BS, blood sample; vas, visual analogue scale; B, block)

To comprehensively assess both subjective and objective dimensions of fatigue and fatigability in individuals with long COVID, the study incorporated three distinct assessment time points (see Fig. 2A): (I) a baseline evaluation scheduled three to four days prior to the stimulation period (pre-assessment), (II) a post-intervention assessment conducted one day after the final tDCS session (post-assessment), and (III) a follow-up assessment one month after the post-assessment to evaluate long-term effects.

As illustrated in Figure 2B, each assessment session began with blood sample collection (not analyzed in the present report), followed by completion of three standardized questionnaires addressing subjective fatigue, depressive symptoms and health-related quality of life (Modified Fatigue Impact Scale, MFIS; Becks Depression Inventory II, BDI-II; European Quality of Life 5 Dimensions 5 Level Version, EQ-5D-5L). Results related to subjective trait fatigue and additional psychometric outcomes are reported elsewhere [58].

After completion of the questionnaires, electroencephalographic (EEG) activity was recorded during the completion of a previously validated fatigue protocol [22, 23]. This protocol began with a paired-click paradigm to assess auditory sensory gating (see below) [22], followed by the administration of a continuous performance task (AX-CPT) designed to induce cognitive fatigue. The session concluded with a second paired-click sequence. Subjective state fatigue was repeatedly assessed throughout the AX-CPT using visual analogue scales (VAS). Objective state fatigue was quantified using both behavioral and electrophysiological markers. Behavioral metrics included response accuracy, mean response times (RT), and standard deviations of RT (SD RT). Concurrent EEG measures focused on frontal theta and occipital alpha power during task performance. Additionally, p50 suppression ratios derived from the paired-click paradigm were analysed. At the end of the follow-up session, participants were asked to indicate whether they believed they had received verum or sham stimulation, in order to assess the integrity of the blinding procedure [69].

### Standard paired click paradigm

To elicit the auditory p50 event-related potential, a standard paired-click paradigm was employed [22]. The protocol consisted of 60 pairs of white-noise clicks (S1 and S2), each with a duration of 1 ms and presented at 80 dB. Prior to stimulus onset, participants were exposed to continuous 30 dB white noise for one minute, which remained active as background noise throughout the procedure. The inter-stimulus interval between the first and second click in each pair was fixed at 500 ms. Between each stimulus pair, the inter-trial interval was jittered randomly between 8 and 11 seconds [22]. The p50 suppression ratio - calculated as the amplitude of the S2 response relative to S1 (s.b.) - served as an index of auditory sensory gating. Changes in suppression ratios across time points within and between assessments were used as a neurophysiological marker of fatigability [22, 27].

### AX-continuous performance task

The AX-Continuous Performance Task (AX-CPT) was utilized to induce cognitive fatigue and simultaneously assess both behavioral performance and subjective state fatigue throughout the task [22, 46]. Stimuli were presented sequentially on a black background and comprised a red cue letter, followed by two white distractor letters, and a red probe letter. Each letter remained on screen for 300 ms, with a fixed inter-stimulus interval of 1200 ms. Participants were instructed to press the right control button as quickly as possible when the red cue “A” was followed by the red probe “X”. For all other combinations, the left control button was used. The task included six blocks of 53 trials each, with two-minute rest intervals between blocks.

Subjective state fatigue was assessed at four time points (queries) during the AX-CPT: Prior to task onset, after blocks two and four, and immediately after task completion. At each point, participants completed two visual analogue scale (VAS) rating their current mental fitness (VAS_fitness_: “How mentally fit do you feel at the moment?”) and their current mental exhaustion (VAS_exhaustion_: “How mentally exhausted do you feel at the moment?”). Responses were recorded on a scale from 0 (“not at all”) to 100 (“extremely”), with the order of questions randomized at each query.

### Gamified Go/No-Go-task

To potentially enhance the effects of stimulation [45, 55], participants engaged in a gamified version of the Go/No-Go paradigm during each tDCS session. The task consisted of ten blocks of 90 trials each, with a fixed Go to No-Go ratio of 2:1. During Go trials, a colored apple (red, yellow, or green) appeared in either the top-left or top-right corner of the screen and descended vertically, simulating a falling motion. The stimulus disappeared once it reached the bottom edge of the display. Participants were instructed to respond as quickly as possible by pressing the left or right control button, corresponding to the apple’s position on the screen. In No-Go trials, a visually similar but bitten apple followed the same falling trajectory, and the participants were instructed to withhold any response. The initial descent duration of the apple was set at 909 ms. Task difficulty was adaptively modulated based on individual performance accuracy across blocks: if accuracy exceeded 90%, stimulus descent speed increased (i.e., duration decreased); if accuracy dropped below 65%, difficulty was reduced by slowing the fall. Accuracy between 65 and 90% resulted in no adjustment. The minimum possible fall duration was limited to 345 ms. At the end of each block, participants received performance feedback. Additionally, auditory warning tones were presented following commission or omission errors. Participants could take self-paced breaks between blocks and resume the task at their own discretion with a button press. The task was completed once daily over four consecutive days. On day one, participants began with the lowest difficulty level. On subsequent days, the starting difficulty was set to two levels below the highest difficulty achieved on the previous day to maintain individual challenge calibration.

### EEG recording and preprocessing

Electroencephalographic (EEG) data were recorded from 23 Ag/AgCl electrodes positioned according to the international 10–20 system mounted in an elastic EEG cap (EasyCap GmbH, Germany). The ground electrode was placed on the forehead, and signals were initially referenced to the nose. Data processing was performed by BrainVision Analyzer 2.2 (BrainProducts, Germany). Offline, all channels were re-referenced to the average of all electrodes. For oscillatory analyses, continuous EEG data was high-pass filtered at 1 Hz, low-pass filtered at 30 Hz and notch filtered at 50 Hz. For p50 analysis, data were high-pass filtered at 0.1 Hz, low-pass filtered at 47 Hz, and notch filtered at 50 Hz to remove line noise. Ocular artifacts were corrected using the Gratton and Coles method [70]. Segments containing voltage steps or peak-to-peak changes exceeding 100 µV within 100 ms were excluded from further analysis.For spectral analyses, EEG data during the AX-CPT was divided into six blocks and segmented into 2-second epochs with a 0.2-second overlap. A Fast Fourier Transformation (FFT) was applied to each epoch using a Hanning window with 10% tapering, yielding a frequency resolution of 0.5 Hz. Spectral power was averaged across epochs within each block. Theta power (4–7.5 Hz) was extracted at electrode Fz, and alpha power (8–12 Hz) was assessed at POz.

To analyze the auditory p50 component, EEG data recorded during the standard click-paradigm were epoched from −150 ms to 450 ms relative to the onset of each auditory stimulus (S1 and S2 seperately) and baseline corrected using an −50 ms to 0 ms interval. S1 and S2 responses were averaged separately. The p50 was identified at electrode Cz. Following the criteria established by Linnhoff et al. [22], amplitude was defined as the voltage difference between the preceding negative deflection and the p50 peak. Sensory gating was quantified using the p50 suppression ratio, calculated as: (1−(S2/S1))*100, with higher values indicating stronger gating. Suppression ratios were computed separately for the pre- and post-AX-CPT measurements. In cases where no discernible S2 response was detected, the amplitude was set to 0.01 µV to enable ratio computation. Suppression ratios exceeding −200% were set to −200% [22]. Five participants were excluded from p50 auditory sensory gating analysis due to the absence of identifiable p50 peaks as defined by Linnhoff et al. during the first auditory gating measurement during the *pre-assessment* [22].

### Data analysis

Data analysis and visualization were performed using RStudio (version 4.3.3; R Core Team, 2024). The success of blinding was evaluated using a chi-square test to compare correct allocation rates between stimulation conditions. Outliers exceeding ±2.5 standard deviations from the mean of each block of each assessment were identified and winsorized to ±2.5 standard deviations in order to reduce the influence of extreme values while preserving the overall structure of the data [71]. Winsorization was applied to all measures of subjective state fatigue, behavioral performance, oscillatory EEG data, and Go/No-Go task variables. Trials classified as invalid, including all error trials, were excluded from the reaction time (RT) analyses. To assess treatment-specific changes in subjective and objective state fatigue, individual time-on-task slopes were computed for subjective ratings, behavioral performance, and electrophysiological measures using the following formula:

$$Slopes = {{\mathop \sum \nolimits_{i = 1}^n \left( {{x_i} - \bar x} \right)\left( {{y_i} - \bar y} \right)} \over {\mathop \sum \nolimits_{i = 1}^n \left( {{x_i} - \bar x} \right)}}$$

In this context, x denotes the measurement time points - either block number (1–6) for behavioral and electrophysiological data or query number (1–4) for VAS-ratings - while y represents the variable of interest expressed in its raw units. In a second step, one-sample t-tests against zero were conducted separately for each experimental group at pre-assessment to determine whether subjective and objective fatigue markers showed significant changes over the course of the task.

Linear Mixed Models (LMMs) were employed in a third step using the *lmer* function from the *afex* package [72]. To account for interindividual variability, random intercepts for subjects were included. Model-III ANOVAs were computed for each proposed model, post hoc comparisons were calculated using the *emeans* package [73]. False discovery rare (FDR) corrections according to Benjamini-Hochberg were applied only to post-hoc comparisons [74], no adjustments were made for omnibus F-tests. In LMMs predicting the slopes of VAS_exhaustion_- and VAS_fitness_-ratings, behavioral performance and EEG frequency data during the AX-CPT *stimulation condition* and *assessment* served as fixed factors. For p50 ratio analyses *stimulation condition*, *assessment* and *time* (begin – end) served as fixed factors to evaluate sensory gating responses across groups. Two participants were unable to attend the follow-up EEG assessment, resulting in 19 participants per stimulation condition at follow-up. Due to additional exclusions in the p50 analysis, the number of valid observations was 16 for the sham and 19 for the verum condition at the pre- and post-assessments and 15 for the sham and 18 for the verum condition at follow-up. LMMs were fitted using all available data, as mixed-effects models handle missing observations under the assumption of missing at random. No additional imputation was applied. There was no missing data in the variables analyzed beyond the reported exclusions. Given the baseline differences, time since infection and WEIMuS cognitive scores were additionally tested as covariates in the LMMs. Neither variable was a significant predictor and the pattern of results remained unchanged.

To evaluate the effects on performance in the gamified Go/No-Go task, LMMs were computed to predict mean difficulty level, accuracy, RTs, SD RTs and d′-prime. Models were based on session-wise means per participant. *Stimulation condition* and *session* were included as fixed effects in all models. To account for the expected influence of task difficulty on accuracy and RTs, *difficulty level* was additionally included as a fixed factor in the models predicting accuracy, RTs, and RT SDs. Random intercepts for the subjects were specified to account for interindividual variability. P-values were FDR-adjusted for all calculations according to the Benjamini-Hochberg procedure [74].

## Results

### Blinding

As previously reported, 70% of participants correctly guessed their stimulation condition in the verum group and 50% in the sham group, with no significant difference in correct allocation rates between conditions (*χ*^*2*^ (1,  = 40) = 1.67, *p* = 0.20, Cramér’s V = 0.20) [58].

### Subjective state fatigue

Time-on-task slopes for VAS_exhaustion_- and VAS_fitness_-ratings were calculated to assess subjective fatigue progression during the AX-CPT. One-sample t-tests revealed significant increases in VAS_exhaustion_ across task duration at baseline in both groups (verum: *t*(19) = 4.78, *p* < 0.001, *d* = 1.07, 95% CI [0.48,1.65]); sham: *t*(19) = 3.11, *p* = 0.006, *d* = 0.69, 95% CI [0.17,1.22]), accompanied by corresponding decreases in VAS_fitness_ (verum: *t*(19) = −2.62, *p* = 0.017, *d* = −0.59, 95% CI [-1.09,-0.08]; sham: *t*(19) = −6.63, *p* < 0.001, *d* = −1.48, 95% CI [-2.16,-0.80]), confirming the fatigability-inducing nature of the AX-CPT.

**Fig. 3:**
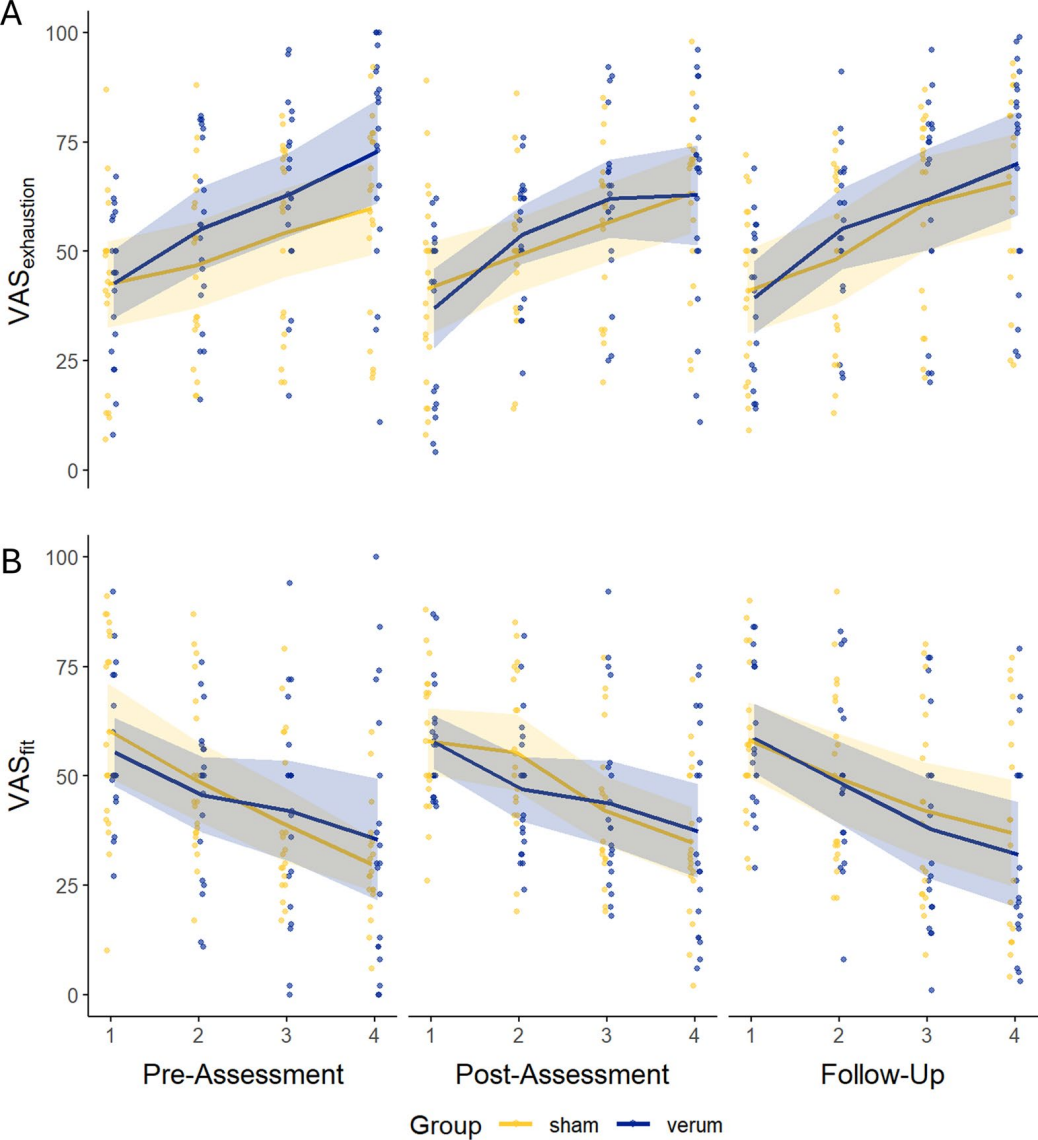
Development of subjective state fatigue over time during the three distinct assessments for a) VAS_exhaustion_-ratings and B) VAS_fitness_-ratings for both stimulation groups; lines represent model-estimated trajectories with 95% confidence intervals, and points show the winsorized raw block-wise data for each participant at each assessment which served as the basis for slope calculation. Higher VAS_exhaustion_-and lower VAS_fitness_-ratings indicate greater levels of cognitive fatigue

VAS-ratings across for the three assessments are displayed in Fig. 3. LMMs were used to examine the effects of stimulation and assessment time point on subjective fatigue trajectories. For VAS_exhaustion_ slopes, no significant main effects were observed for *assessment time point* (*F*(2, 75.02) = 0.57, *p* = 0.567, *η*^*2*^_*p*_ = 0.02), *stimulation condition* (*F*(1, 38.44) = 1.12, *p* = 0.296, *η*^*2*^_*p*_ = 0.03), or their interaction (*F*(2, 75.02) = 0.48, *p* = 0.623, *η*^*2*^_*p*_ = 0.01). Similarly, for VAS_fitness_- slopes, there were no significant main effects of *assessment time point* (*F*(2, 74.24) = 0.29, *p* = 0.752, *η*^*2*^_*p*_ = 0.01), *stimulation condition* (*F*(1, 37.67) = 0.35, *p* = 0.560, *η*^*2*^_*p*_ = 0.01), or their interaction (*F*(2, 74.24) = 2.48, *p* = 0.091, *η*^*2*^_*p*_ = 0.06). No alleviating effects of tDCS on the development of subjective state fatigue were observed

### Objective state fatigue

#### Behavioral measurements

LMMs of behavioral performance during the AX-CPT revealed no significant time-on-task effects at baseline in either the verum or sham group (sham: *t*(19) = −1.25, *p* = 0.226, *d* = −0.28, 95% CI [-0.75,0.20]; verum: *t*(19) = −2.02, *p* = 0.115, *d* = −0.45, 95% CI [-0.94,0.04]), RTs (sham: *t*(19) = −0.92, *p* = 0.371, *d* = −0.21, 95% CI [-0.68,0.27]; verum: *t*(19) = −0.96, *p* = 0.350, *d* = −0.21, 95% CI [-0.67,0.26]) or RT SDs (sham: *t*(19) = 1.35, *p* = 0.192, *d* = 0.30, 95% CI [-0.18,0.78]; verum: *t*(19) = 0.66, *p* = 0.518, *d* = 0.15, 95% CI [-0.32,0.62]). No systematic changes were observed in the temporal trajectories of accuracy, RTs and RT SDs across the six task blocks, indicating that behavioral parameters were not sensitive to experimental fatigue induction.

LMMs predicting behavioral performance during the AX-CPT revealed no significant main effects or interactions involving stimulation condition or assessment time point on behavioral state fatigue markers, with no evidence of tDCS-related effects (see Table 3).

**Table 3:** ANOVA-results of the LMMs predicting changes of behavioral outcomes during the AX-CPT

Behavioral Parameter	Predictors	F	df1, df2	p	partial η^2^
Accuracy	Stimulation Condition	1.53	1,38.41	0.224	0.03
	Assessment Time-Point	2.26	2,75.30	0.113	0.05
	Stimulation Condition × Assessment Time-Point	0.11	2,75.31	0.732	0.01
Reaction Times	Stimulation Condition	0.18	1,38.48	0.671	0.00
	Assessment Time-Point	0.38	2,75.58	0.686	0.01
	Stimulation Condition × Assessment Time-Point	0.13	2,75.58	0.879	0.00
Standard Deviations of RTs	Stimulation Condition	0.33	1,37.88	0.570	0.01
	Assessment Time-Point	0.52	2,75.05	0.560	0.01
	Stimulation Condition × Assessment Time-Point	1.12	2,75.05	0.335	0.03

#### Electrophysiological objective state fatigue

**Fig. 4:**
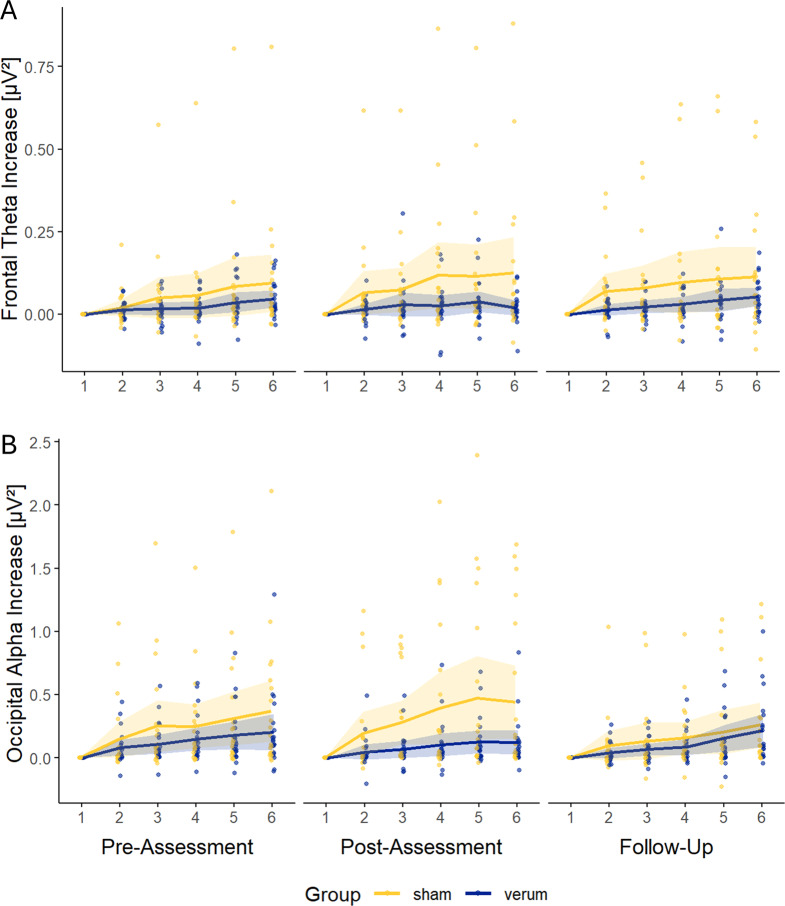
Alterations to baseline (block 1) of a) frontal theta and B) occipital alpha activity over time on task for both stimulation groups; lines represent model-estimated trajectories with 95% confidence intervals, and points show the winsorized raw block-wise data for each participant at each assessment which served as the basis for slope calculation. For visualization purposes only, all values were normalized to block 1 (normalized value = blockᵢ – block₁)

Alterations of frontal theta and occipital alpha activity are depicted in Fig. 4. At baseline, frontal theta power increased over the course of the AX-CPT in both groups (verum: *t*(19) = 3.52, *p* = 0.002, *d* = 0.79, 95% CI [0.25,1.32]; sham: *t*(19) = 2.22, *p* = 0.039, *d* = 0.50, 95% CI [0.00,0.99]). However, the LMM revealed no significant main effects for *assessment time-point* (*F*(2,74.28) = 0.06, *p* = 0.938. *η*^*2*^_*p*_ = 0.00), *stimulation condition* (*F*(1,38.13) = 2.38, *p* = 0.131, *η*^*2*^_*p*_ = 0.06), or their interaction (*F*(2,74.28) = 1.60, *p* = 0.208, *η*^*2*^_*p*_ = 0.04) on the slope of frontal theta power.

Analogously, occipital alpha power increased over the course of the AX-CPT at baseline in both groups (verum: *t*(19) = 3.33, *p* = 0.004, *d* = 0.74, 95% CI [0.22,1.27]; sham: *t*(19) = 3.38, *p* = 0.004, *d* = 0.76, 95% CI [0.23,1.29]). The LMM revealed no significant main effect of *assessment time-point* (*F*(2, 74.45) = 1.04, *p* = 0.360, *η*^*2*^_*p*_ = 0.03) or *stimulation condition* (*F*(1, 38.18) = 2.28, *p* = 0.140, *η*^*2*^_*p*_ = 0.06) on occipital alpha slopes. However, the LMM revealed a significant *stimulation* × *assessment* interaction (*F*(2, 74.45) = 4.18, *p* = 0.020, *η*^*2*^_*p*_ = 0.10). Post hoc comparisons indicated a stronger increase in occipital alpha power at *post-assessment* in the sham group compared to the verum group (mean difference = 0.06, *p* = 0.011, *d* = 0.33, 95% CI [0.07,0.59]), as well as a reduction in alpha increase from post-assessment to follow-up in the sham group (mean difference = 0.05, *p* = 0.010, *d* = 0.35, 95% CI [0.11,0.58]).

Analysis of p50 suppression ratios revealed no significant main effects for *time* (*F*(1, 156.65) = 3.79, *p* = 0.054, *η*^*2*^_*p*_ = 0.02), *assessment time-point* (*F*(1, 158.30) = 0.91, *p* = 0.402, *η*^*2*^_*p*_ = 0.01), or *stimulation condition* (*F*(1, 28.92) = 0.97, *p* = 0.333, *η*^*2*^_*p*_ = 0.03). Furthermore, none of the interaction terms reached statistical significance (all *p*s > 0.05). However, trend-level effects were observed for the interaction of *time* and *stimulation condition* (*F*(1, 156.65) = 3.72, *p* = 0.056, *η*^*2*^_*p*_ = 0.02), resulting from a significant p50 suppression ratio difference before and after the AX-CPT solely in the sham (mean difference = 29.90, *p* = 0.010, *d* = 0.21, 95% CI[0.05,0.36]), but not in the verum group (mean difference = 0.13, *p* = 0.990, *d* = 0.00, 95% CI[−0.15,0.16]). This could suggest a baseline group difference in reduction in sensory gating following fatigue induction across assessments.

#### Gamified Go/No-Go task

The LMM predicting task difficulty revealed no significant main effect of *stimulation condition* on difficulty level (*F*(1, 38) = 0.01, *p* = 0.912, *η*^*2*^_*p*_ = 0.00). However, a significant main effect of *stimulation session* was observed (*F*(3, 114) = 142.93, *p* < 0.001, *η*^*2*^_*p*_ = 0.79), reflecting progressive increases in task difficulty across sessions. No significant *stimulation condition* × *session* interaction was observed (*F*(3, 114) = 1.40, *p* = 0.248, *η*^*2*^_*p*_ = 0.04). Post hoc comparisons confirmed significant increases in difficulty from stimulation session 1 to sessions 2, 3 and 4 (mean difference 1–2 = 2.11, *p* < 0.001, *d* = 1.33, 95% CI[1.07,1.58]; mean difference 1–3 = 2.54, *p* < 0.001, *d* = 1.59, 95% CI[1.31,1.87]; mean difference 1–4 = 2.72, *p* < 0.001, *d* = 1.71, 95% CI[1.42,2.00]), as well as significant increases between stimulation sessions 2 and 3 (mean difference = 0.43, *p* = 0.005, *d* = 0.27, 95% CI[0.08,0.46]) and 2 and 4 (mean difference = 0.61, *p* < 0.001, *d* = 0.39, 95% CI[0.19,0.58]).

The LMM predicting accuracy during the gamified Go/No-Go task revealed a significant main effect of *stimulation session* (*F*(3, 109.15) = 6.47, *p* < 0.001, *η*^*2*^_*p*_ = 0.15) and a significant *stimulation session × difficulty* interaction (*F*(3, 112.00) = 9.08, *p* < 0.001, *η*^*2*^_*p*_ = 0.20). Interactions at trend level were observed between *stimulation condition × session* (*F*(3, 109.15) = 2.62, *p* = 0.055, *η*^*2*^_*p*_ = 0.07), as well *as stimulation condition × stimulation session × difficulty* (*F*(3, 112.00) = 2.61, *p* = 0.055, *η*^*2*^_*p*_ = 0.07). No significant effects were found for *stimulation condition, difficulty* or the interaction of *stimulation condition* and *difficulty* (all *p*s > 0.2).

Post hoc tests examining the simple slopes of *difficulty* across sessions revealed a significantly positive effect of difficulty on accuracy in *stimulation session 1* (*b* = 0.0092, *p* = 0.003, *d* = 0.26, 95% CI[0.08,0.43]), but significantly negative effects in *stimulation session 2* (*b* = −0.0042, *p* = 0.022, *d* = −0.20, 95% CI[−0.38,-0.03]) and *4* (*b* = −0.0051, *p* = 0.014, *d* = −0.21, 95% CI[−0.39,-0.04]). No significant effect of *difficulty* on accuracy was found in *stimulation session 3* (*b* = −0.0002, *p* = 0.920, *d* = −0.01, 95% CI[−0.18,0.16]).

The model predicting RTs during the gamified Go/No-Go task revealed a significant main effect of *stimulation session* (*F*(3, 97.20) = 5.75, *p* = 0.001, *η*^*2*^_*p*_ = 0.15), a main effect of *difficulty* (*F*(1, 117.02) = 61.16, *p* < 0.001, *η*^*2*^_*p*_ = 0.34), and a significant *stimulation session × difficulty* interaction (*F*(3, 100.65) = 3.36, *p* = 0.022, *η*^*2*^_*p*_ = 0.09). No effects involving *stimulation condition* reached significance (all *p*s > 0.2). Post hoc tests regarding the interaction of *stimulation session* x *difficulty* revealed a stronger negative impact of task difficulty in *stimulation session 1* compared to *session 4* (mean difference = 15.19, *p* = 0.013, *d* = −0.29, 95% CI[−0.47,-0.10]). Descriptive differences regarding the influence of task difficulty on RTs where also found between *stimulation sessions 1 and 2* (mean difference = 10.23, *p* = 0.063, *d* = −0.20, 95% CI[−0.40,-0.02]) and *1 and 3* (mean difference = 11.25, *p* = 0.058, *d* = −0.22, 95% CI[−0.41,-0.03])

The LMM predicting SD RTs during the Go/No-Go-task revealed a significant main effect of *difficulty* (*F*(1, 48.42) = 79.86, *p* < 0.001, *η*^*2*^_*p*_ = 0.61). No other significant main effects or interactions where found (all *p*s > 0.1). A post hoc analysis of the effect of difficulty on RT SDs revealed a generally enhancing effect of *difficulty* (*b* = −7.84, *p* < 0.001, *d* = −1.14, 95% CI[-1.48,-0.81]).

The LMM predicting *d*′-prime values during the gamified Go/No-Go task revealed a significant main effect of *stimulation session* (*F*(3, 110.02) = 3.56, *p* = 0.017, *η*^*2*^_*p*_ = 0.09) and *stimulation condition* (*F*(1, 49.00) = 6.22, *p* = 0.016, *η*^*2*^_*p*_ = 0.11). Further significances were found for the interactions of *stimulation session × condition* (*F*(3, 110.20) = 5.57, *p* = 0.001, *η*^*2*^_*p*_ = 0.13), *stimulation session × difficulty* (*F*(3, 111.83) = 16.41, *p* < 0.001, *η*^*2*^_*p*_ = 0.31) and *stimulation condition × session × difficulty* (*F*(3, 111.85) = 3.93, *p* = 0.010, *η*^*2*^_*p*_ = 0.10). Other effects did not reach significance levels (all *p*s > 0.08).

Post hoc tests regarding the three-way interaction revealed a significant positive effect of *task difficulty* on *d*′-prime values in both stimulation conditions during the first session (verum: *b* = 0.46, *p* < 0.001, d = 0.44,95% CI[0.27,0.61]; sham: *b* = 0.32, *p* = 0.001, d = 0.27, 95% CI[0.10,0.44]). In contrast, significant negative effects of difficulty on *d*′-prime were found in the verum group during *stimulation sessions 3* and *4* (verumxsession3: *b* = −0.18, *p* < 0.001, *d* = −0.29, 95% CI[-0.46,-0.12]; verumxsession4 = −0.19, *p* = 0.004, *d* = −0.25, 95% CI[-0.41,-0.08]). No other *stimulation condition × session* combinations showed significant effects of task difficulty on *d*′-prime.

## Discussion

This preregistered, double-blind, randomized controlled trial investigated the effects of four consecutive daily sessions of anodal tDCS over the left dlPFC on cognitive state fatigue in individuals with long COVID associated fatigue. The present report focuses on the secondary outcomes addressing subjective and objective parameters of state fatigue. Specifically, we examined within-task changes in variables reflecting subjective experience, objective performance, and electrophysiological alterations.

In sum, this preregistered trial revealed that subjective state fatigue ratings as well as EEG frontal theta and occipital alpha power increased over time-on-task, indicating successful induction of state fatigue and increased cognitive effort during task engagement. However, these effects were largely invariant across assessments and were not modulated by stimulation condition. Similarly, behavioral performance metrics as well as sensory gating showed no evidence of stimulation-related or assessment-dependent modulation. A notable exception emerged in occipital alpha activity, where a significant interaction indicated a more pronounced post-intervention increase in the sham group relative to the verum group. Taken together, these findings suggest that four consecutive sessions of tDCS over the left dlPFC, administered during a gamified Go/No-Go task, do not induce generalized improvements in state fatigue in individuals with long COVID related cognitive fatigue. More robust outcomes may require alternative stimulation protocols, including extended treatment durations, or cognitively more demanding task paradigms. However, even in the absence of robust treatment effects, the current findings offer valuable insights into the neurophysiological profile of fatigue and inform future intervention strategies by delineating boundary conditions for tDCS efficacy.

The present study confirmed the AX-CPT as a robust and suitable paradigm for inducing cognitive state fatigue in individuals with long COVID, as evidenced by consistent time-on-task effects across subjective and neurophysiological measures. Participants consistently reported progressively increasing mental exhaustion over the course of the task, as reflected in the elevation of VAS ratings of cognitive exhaustion and corresponding declines in perceived cognitive fitness. These systematic within-task shifts in subjective fatigue markers underscore the AX-CPT’s validity as a cognitively demanding task in this clinical population.

Electrophysiological frequency-band measures likewise demonstrated sensitivity to the emergence of cognitive state fatigue. Frontal theta and occipital alpha activity increased over the course of the task, paralleling subjective state fatigue trajectories. In contrast, behavioral performance remained stable across time and showed no sensitivity to fatigue induction. No differential effects between stimulation groups were observed. Despite increasing subjective state fatigue, performance remained stable. This dissociation between subjective and behavioral indices may reflect compensatory engagement of cognitive control resources, enabling participants to maintain performance despite rising state fatigue [18]. Although reaction time variability has been proposed as a sensitive marker of attentional fluctuations in long COVID patients [75, 76], the AX-CPT may have been too simple and slow-paced to elicit reliable behavioral decline, potentially obscuring stimulation-related effects due to ceiling effects [77]. However, these findings suggest that spectral EEG markers may hold greater sensitivity than behavioral metrics to detect early or subtle manifestations of state fatigue. It is conceivable that participants maintained behavioral performance through compensatory engagement of cognitive control processes, as indexed by increasing frontal theta activity [24].

Importantly, while theta-band responses remained unaffected by stimulation, occipital alpha activity showed a differential modulation. The increase of occipital alpha power enhanced significantly from pre- to post-assessment in the sham group whereas it remained stable in the verum group, suggesting a potential neurophysiological effect of the tDCS. Notably, this alpha increase in the sham group was transient and returned to baseline at follow-up, in contrast to more persistent reductions in trait fatigue reported elsewhere. [58] The pattern of results suggests that tDCS may have helped to maintain occipital alpha reactivity over time, although no stimulation effects were evident in behavioral performance or subjective fatigue measures. Although the findings are encouraging, the observed interaction should be interpreted with caution given that multiplicity correction was limited to post-hoc tests. In contrast, the absence of tDCS-related modulation in frontal theta power may reflect slower or more cumulative effects of stimulation [25], or alternatively, indicate that more prolonged stimulation protocols or higher cognitive demands are necessary to elicit measurable changes in this frequency band.

Finally, the p50 analysis did not reveal any stimulation-specific effects. However, descriptive group differences in p50 suppression ratios emerged following the fatiguing task. Such differences may reflect variability inherent to the between-subjects design used in this study. Additionally, five participants had to be excluded due to absent or invalid electrophysiological responses, resulting in unequal group sizes and reducing the statistical power of the analysis. These methodological constraints limit the interpretability of the observed effects and highlight the challenges associated with using p50 suppression as a reliable marker in clinical fatigue research. Nevertheless, the current findings underscore the potential relevance of p50 sensory gating in capturing fatigue-related alterations in early pre-attentive processing, as previously suggested in the context of both neurological and post-viral fatigue syndromes [10, 22]. Future studies employing within-subject crossover designs, larger sample sizes, and optimized data acquisition protocols may help to clarify the sensitivity and specificity of p50 suppression as a neurophysiological correlate of cognitive fatigability in long COVID.

No direct effects of tDCS on performance in the gamified Go/No-Go task were observed. Nonetheless, participants in both groups demonstrated adaptive learning effects across sessions, despite elevated levels of trait fatigue [58]. Accuracy initially benefited from increasing task difficulty but declined in later sessions, while overall performance remained high, likely due to the task’s adaptive design. Reaction times and their variability also improved across sessions. Notably, d′-prime values revealed a more nuanced pattern: task difficulty improved d′-prime in the first session across both groups, but in the verum group, this effect reversed in later sessions. This shift may reflect complex interaction between stimulation-induced cortical excitability and evolving task demands, particularly in cognitive domains such as response inhibition, which are critically reliant on prefrontal integrity [78–80]. Although structural and functional alterations of the frontal cortex have been reported in individuals with long COVID [28] participants maintained high d′-prime values throughout, suggesting preserved global detection performance [81]. Future research should continue to explore the complex interplay between subjective state fatigue, its behavioral and neurophysiological correlates, and domain-specific cognitive profiles in long COVID.

Interpretation of these findings is constrained by some limitations. The study was powered to detect changes in subjective trait fatigue which was defined as the primary outcome and not specifically to detect fatigue trajectories during time-on-task that were defined as secondary outcomes. Additionally, unlike the electrophysiological measures, the behavioral parameters used in this protocol were not sensitive to the experimental fatigue induction and may have exhibited a ceiling effect, further limiting the detectability of stimulation effects. Finally, because only post-hoc tests were corrected for multiplicity, the findings warrant cautious interpretation. Despite randomization, groups differed in their time since acute SARS-CoV-2 infection, which could have a modulating effect on subjective exhaustion, objective fatigue outcomes and responsiveness to stimulation. Therefore, the modest sample size, between-subjects design, and exclusion of severely affected individuals - due to the risk of post-exertional malaise (PEM) - reduced statistical power and generalizability. PEM remains a central challenge in long COVID research [82]. To mitigate risks, only mildly to moderately affected individuals were included, potentially limiting ecological validity. Future studies should employ crossover designs, larger samples, and explore telemedical tDCS approaches to enhance accessibility and inclusivity [83].

## Conclusions

In conclusion, while anodal tDCS over the left dLPFC did not produce consistent improvements in subjective or behavioral indicators of cognitive state fatigue, electrophysiological findings suggest a potential stabilizing effect on cortical excitability, as reflected in attenuated occipital alpha reactivity. These results reinforce the importance of including neurophysiological endpoints in intervention research and highlight the need for more robust and individualized stimulation protocols. Future studies should seek to delineate the dynamic interplay between subjective fatigue, neurophysiological markers, and cognitive performance in long COVID, thereby refining strategies for managing this complex and heterogeneous condition.

## Data Availability

Data sets generated are available from the corresponding author upon reasonable request

## Abbreviations

tDCS: Transcranial direct current Stimulation
dlPFC: Dorsolateral prefrontal cortex
MS: Multiple Sclerosis
ME/CFS: Myalgic Encephalitis/Chronic Fatigue Syndrome
LMM: Linear Mixed Model
AX-CPT: AX-Continuous Performance Task
RT: Reaction Time
SD RT: Standard Deviation of Reaction Time
PEM: Post-exertional Malaise
